# FRAILTY STATUS AND CATHETER ABLATION THERAPY FOR ATRIAL FIBRILLATION IN REAL-AF: A MULTICENTER CLINICAL REGISTRY

**DOI:** 10.1093/geroni/igae098.1453

**Published:** 2024-12-31

**Authors:** Paulo Chaves, Allyson Varley, Anil Rajendra, Dinesh Sharma, Christopher Thorne, Paul Zei, Jose Osorio

**Affiliations:** Florida International University, Miami, Florida, United States; HRCRS, Birmingham, Alabama, United States; Grandview Medical Center, Birmingham, Alabama, United States; NCH Health Care System, Naples, Florida, United States; Heart Rhythm Clinical Research Solutions, Birmingham, Alabama, United States; Brigham and Women’s Hospital, Boston, Massachusetts, United States; HCA Florida Miami, Miami, Florida, United States

## Abstract

There has been increasing interest in understanding if frailty assessment could improve clinical decision-making related to catheter ablation therapy (CAT) for atrial fibrillation (AF) patients. This prospective study assessed acute hospital complications, AF recurrence and AF-related hospitalization within 12 months after CAT according to frailty status pre-CAT. Study subjects (n=3,406) were patients in Real-AF, a multicenter clinical registry in the US, who underwent CAT with 12-month outcome data (2018-2022). Frailty scores were derived using the cumulative health deficit approach (deficits count divided by the total number of health indicators), and categorized: Fit (≤.12), Intermediate (.12–.24), and Frail (≥0.25). Cross-tabulations, trend tests, and logistic regression were performed. Mean age± SD of study subjects was 65.2± 11.0; males were 59.0%. Among patients who underwent CAT, 24.5% met criteria for Frail (score≥0.25). Frailty scores were correlated weakly with age (r=0.09; p<.01). Fit (1.2%) and Frail (1.3%) had similar acute in-hospital complication rates. Increasingly higher probability of AF recurrence (18.2% vs. 19.8% vs. 22.6%; p=.02), and AF-related hospital admission (2.1% vs. 3.4% vs. 4.6% vs. 5.4%, p=.02) within 12 months after CAT were observed across increasing frailty severity categories. Frailty remained associated with AF recurrence and hospitalization in age- and sex-adjusted models; e.g., odds of hospitalization post-CAT were 2.3 (95%CI: 1.0-5.0, p=.039) times higher in Frail than in Fit. Despite adverse trends with frailty, absolute 12-month risks after CAT, even among Frail, was low. Implementation research is warranted to establish the clinical value of frailty assessment for CAT-related decision-making in AF patients.

